# Supplementary figures and images for: Ecdysone-controlled nuclear receptor ERR regulates metabolic homeostasis in the disease vector mosquito Aedes aegypti
Source: PLoS Genet. 2024 Mar 11;20(3):e1011196. doi: 10.1371/journal.pgen.1011196 (PMC10957079; doi:10.1371/journal.pgen.1011196)

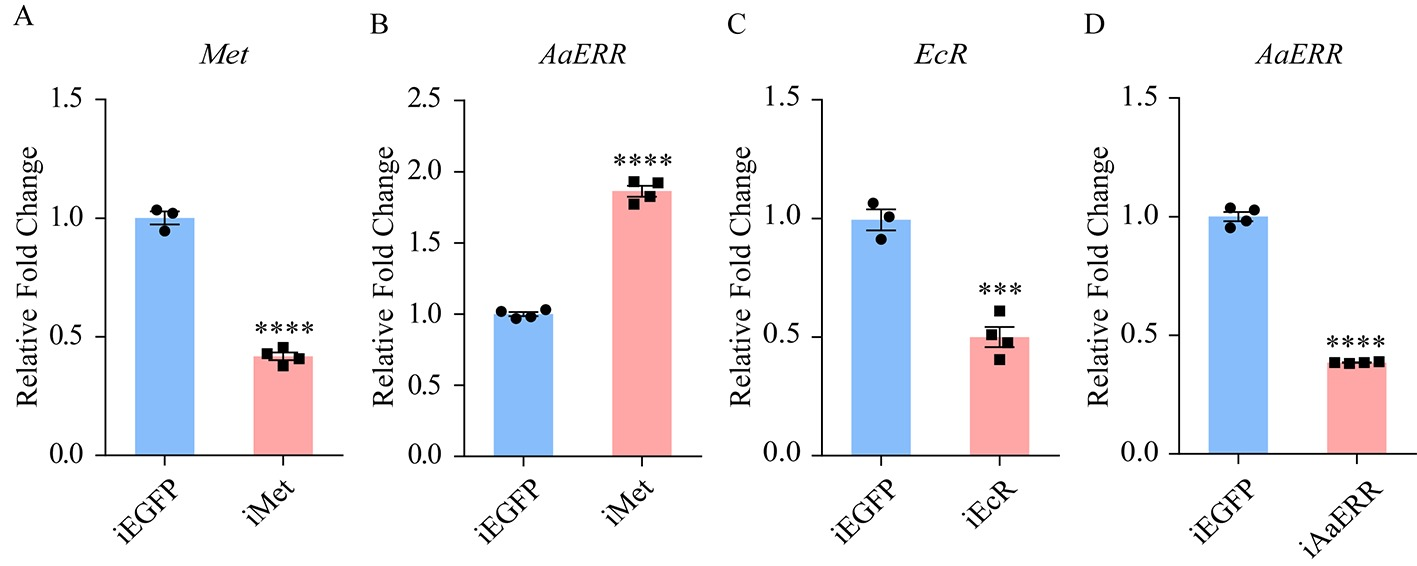

Supplement: S1 Fig — The relative mRNA levels of Met (A) and AaERR (B) after Met knockdown (Two-tailed Unpaired t test: ****p < 0.0001; at least three biological replicates). Error bars are shown as mean ± SEM. (C) The relative mRNA level of EcR after EcR knockdown (Two-tailed Unpaired t test: ****p = 0.0005; at least three biological replicates). Error bars are shown as mean ± SEM. (D) The relative mRNA level of AaERR after AaERR knockdown (Two-tailed Welch’s t test: ****p < 0.0001; at least three biological replicates). Error bars are shown as mean ± SEM. EGFP knockdown was used as the control in (A-D). (TIF) [file pgen.1011196.s001.tif]

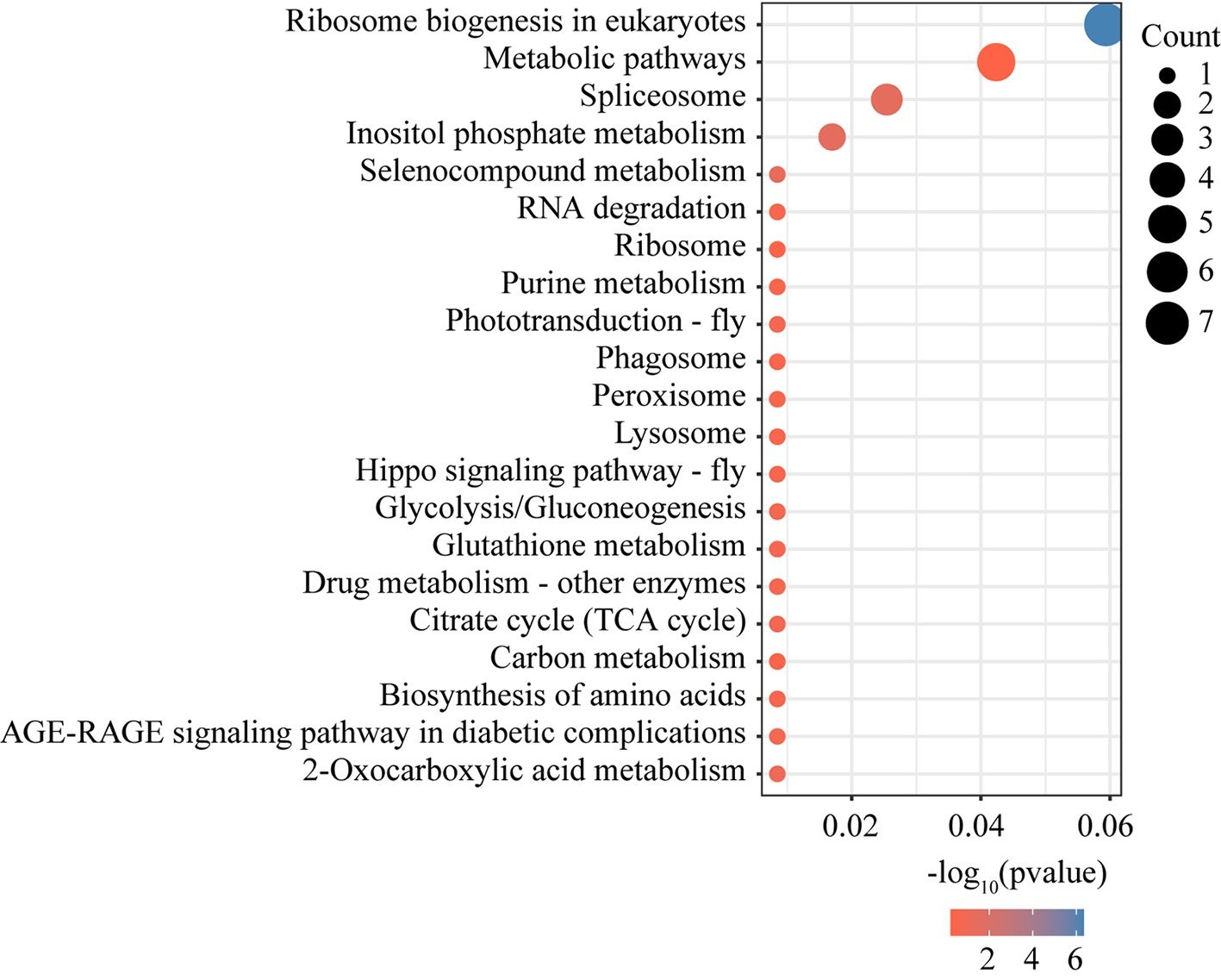

Supplement: S2 Fig — KEGG pathway enrichment analysis was performed on the upregulated DEGs in FBs of iAaERR female mosquitoes. (TIF) [file pgen.1011196.s002.tif]

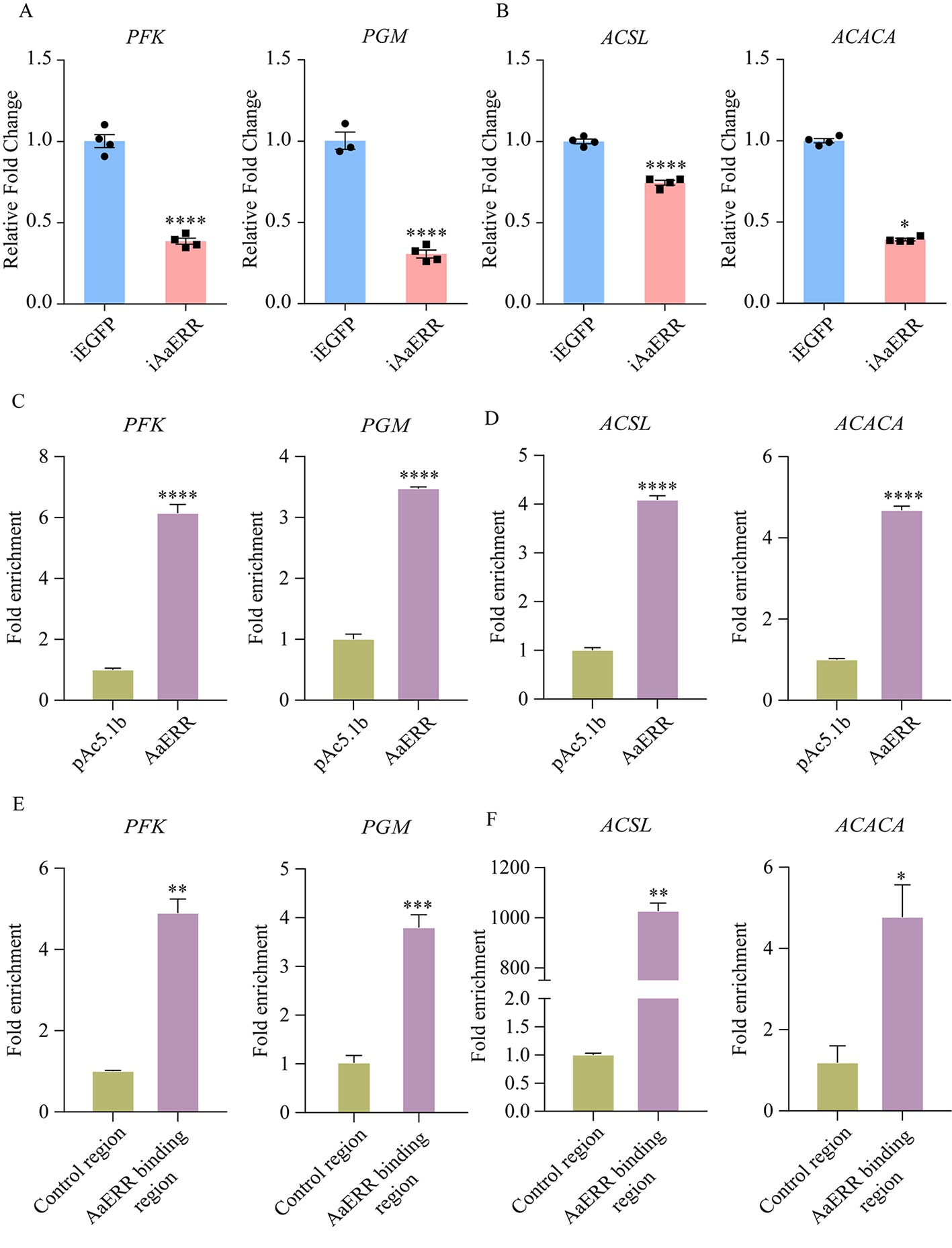

Supplement: S3 Fig — (A-B) The relative mRNA levels of CM and LM genes were analyzed using qRT-PCR in iAaERR mosquitoes (Two-tailed Unpaired t test: ****p < 0.0001; Two-tailed Mann-Whitney test: *p = 0.0286; at least three biological replicates). Error bars are shown as mean ± SEM. PFK, phosphofructokinase; PGM, phosphoglucomutase; ACSL, long-chain acyl-CoA synthetase; ACACA, acetyl-CoA carboxylase/biotin carboxylase 1. iEGFP samples were used as control. (C-D) ChIP-qPCR analysis using anti-V5 and anti-IgG antibodies assessed the enrichment of PFK, PGM, ACSL, and ACACA promoter fragments in Aag2 cells transfected with pAc5.1b-AaERR-V5. Control cells were transfected with the pAc5.1b vector and subjected to the same antibody analysis. The graph illustrated the relative fold enrichment for promoter fragments of each gene (Two-tailed Unpaired t test: ****p < 0.0001; three biological replicates). Error bars are shown as mean ± SEM. (E-F) ChIP with an anti-V5 antibody followed by qPCR assessed the enrichment of the regulatory regions versus control regions within the coding sequences of PFK, PGM, ACSL, and ACACA. (Two-tailed Welch’s t test: **p = 0.0075 (PFK); Two-tailed Unpaired t test: ***p = 0.0008 (PGM); Two-tailed Welch’s t test: **p = 0.001 (ACSL); Two-tailed Unpaired t test: *p = 0.0162 (ACACA); three biological replicates). Error bars are shown as mean ± SEM. (TIF) [file pgen.1011196.s003.tif]
