# Supplementary material for: Ecdysone-controlled nuclear receptor ERR regulates metabolic homeostasis in the disease vector mosquito Aedes aegypti
Source: PLoS Genet. 2024 Mar 11;20(3):e1011196. doi: 10.1371/journal.pgen.1011196 (PMC10957079; doi:10.1371/journal.pgen.1011196)
Supplement: S1 Table — (DOCX) [file pgen.1011196.s004.docx]

**S1 Table. The primers used in this study.**

| **Gene name** | **Forward primers (5'-3')** | **Reverse primers (5'-3')** | **Primer use** |
| --- | --- | --- | --- |
| AaERR | TCGTAGAGATGCTGGAATCG | GTGAGCCTGAGCACCTACAA | qRT-PCR |
| EcR | GCACAGGGACAGCAGGTGAT | ATCGTGGTAGCATTGAGGGC | qRT-PCR |
| Met | GGACGACAGCTCAAAGAAGG | TCACTTTCATCGGGGAGTTC | qRT-PCR |
| Vg | CCAGAAGACGTGAGCATTCA | TGGCGCAGATGATAGAACAG | qRT-PCR |
| GPI | GTATCGGTGGTTCGGATTTG | CAGCTTCTTCAAGGTTTCCG | qRT-PCR |
| PYK | CGTTGAACAGGGTGTTGATG | CCTGCTGGTTCTCGATCTTC | qRT-PCR |
| PGM | AAGGACGGAGTGGTTCACAC | ATGTCGTAATCTCCGTTCCG | qRT-PCR |
| PFK | GCCGGTCAAAGATTGAACAT | AGCACCGTAATCCTGGTGTC | qRT-PCR |
| FAS | TGCTTCCGATTCGCGCCGTA | TCAGGCGCTTCTGCATGGCT | qRT-PCR |
| ACSL | TGCTACTCAGGATGGCACTGAACG | AGCGTTACACGAAACTGGTGGACC | qRT-PCR |
| ACACA | GGCAAGATCGGTTTTCCGGTGATGA | ATCTTCGAAGACTTCAGGCTCTGCA | qRT-PCR |
| EGFP | T7-GGCAAGCTGACCCTGAAGTT | T7-GTCCATGCCGAGAGTGATCC | dsRNA |
| AaERR | T7-AAACAAGAGGCGGCGAAAA | T7-GGAGTGAGGGCAGGAGCAA | dsRNA |
| EcR | T7-CGGAGCGTCACCAAGAATG | T7-TGGCGAACTCCACGATTAG | dsRNA |
| AaERR-pAc5.1b | GTGTGGTGGAATTCTGCAATGATGGACAGTTGGATG | GCCACTGTGCTGGATATCTCGCGATACCGATTCCAG | Gene clone |
| AaERR-pGL4.10 | CCGGTACCTGAGCTCGCTAATGTTGGTGCTACAAAGAC | CTTGATATCCTCGAGGCT ACTAAATGGGAGAAGGGAAG | Gene clone |
| GPI-pGL4.10 | CCGGTACCTGAGCTCGCTCAGTGGATGAACTCTTGTGGGA | CTTGATATCCTCGAGGCTACGACACCACACACAAAAGACG | Gene clone |
| PYK-pGL4.10 | CCGGTACCTGAGCTCGCTCGCACCGTTGCAGAAATTGTTCTC | CTTGATATCCTCGAGGCTACTCACTTCCACTCGGCTGCTTT | Gene clone |
| FAS-pGL4.10 | CCGGTACCTGAGCTCGCTGGTGGGAAACTTGAGTTGATTCTAGCG | CTTGATATCCTCGAGGCTGGAGCTCCGAAATGTGCATGGG | Gene clone |
| pGL4.10 | AGCCTCGAGGATATCAAG | AGCGAGCTCAGGTACCGG | Vector linearization |
| pAc5.1b | GATATCCAGCACAGTGGC | TGCAGAATTCCACCACAC | Vector linearization |
| AaERR | ATGTTTAAGTGCTCAATGAACTTTCACCTT | AAGGTGAAAGTTCATTGAGCACTTAAACAT | EMSA |
| GPI | AGACATAAGAAAAAGGTCAGTAGAAAAAAG | CTTTTTTCTACTGACCTTTTTCTTATGTCT | EMSA |
| PYK | CTGACATACCCTAAGGTCCCAAAAAAACGA | TCGTTTTTTTGGGACCTTAGGGTATGTCAG | EMSA |
| FAS | TGTAGTTTGCAAAGGTCATATCGTAACTAT | ATAGTTACGATATGACCTTTGCAAACTACA | EMSA |
| AaERR | CTGTCGGTGGAGGTATACATGGG | GAGAATTGGACCAGCCTTGGGGA | ChIP-qPCR (EcR binding region in *AaERR* promoter) |
| AaERR | GCGTGTCAAGCCTGCCGATTC | GCGGTGTGTACTGCGAACTGGA | ChIP-qPCR (*AaERR* coding region as a control) |
| GPI | GTGGAGAAGTATAGTCACACAGGAC | AATACTAAGCAAGATGCTCTGTAGC | ChIP-qPCR (AaERR binding region in *GPI* promoter) |
| GPI | GAGCGGTGCTGCATATTGCGC | CCGATGTTGACCACATCGCTGAT | ChIP-qPCR (*GPI* coding region as a control) |
| PYK | CGCTCTGCGTTCTGATTTAGTGCC | TTGCTTGGATTGAATGGAGACGG | ChIP-qPCR (AaERR binding region in *PYK* promoter) |
| PYK | TACTGGGCTGATCGAGGGCAG | TCGACGAACACCCTATCGCCC | ChIP-qPCR (*PYK* coding region as a control) |
| PFK | CCTTGCAGTATTCCCTGAGAAATTT | CCAGCTGAATTTCCTCCGCAATAT | ChIP-qPCR (AaERR binding region in *PFK* promoter) |
| PFK | CCCGCTGCATGGACTTCAAGG | GTGCGCTGGTCCTGAGTGATC | ChIP-qPCR (*PFK* coding region as a control) |
| PGM | GTTGCCTCTAGTTGTGGATCGAG | TGCAGGGCTGTTACAAAAACAAACG | ChIP-qPCR (AaERR binding region in *PGM* promoter) |
| PGM | GGATCGTCATGACCGCTTCCC | CCCTTCTGCGATCTTGTACTCC | ChIP-qPCR (*PGM* coding region as a control) |
| FAS | CGTTCCTCCAAGTCTAATGCTTCC | GACATAATACGTTCCGGTGCCATC | ChIP-qPCR (AaERR binding region in *FAS* promoter) |
| FAS | CTACGATGGCTGTCAGATTCAGG | TGGTCGGGAGGTCGTACAATCC | ChIP-qPCR (*FAS* coding region as a control) |
| ACSL | CTGGGAAAATTGCAGTTCTCACTG | TCGTCGTATAAGCAAATCGGCGC | ChIP-qPCR (AaERR binding region in *ACSL* promoter) |
| ACSL | TGCTACTCAGGATGGCACTGAACG | AGCGTTACACGAAACTGGTGGACC | ChIP-qPCR (*ACSL* coding region as a control) |
| ACACA | TGACCCTACTCTCGATTCCAGAG | CGAAAGAAGCATCAATGACAGTGC | ChIP-qPCR (AaERR binding region in *ACACA* promoter) |
| ACACA | TGCTATCGATGCCGTTGCGCAG | GGAATCCATTCCGTTCCCAGCA | ChIP-qPCR (*ACACA* coding region as a control) |
