# Supplementary material for: Ecdysone-controlled nuclear receptor ERR regulates metabolic homeostasis in the disease vector mosquito Aedes aegypti
Source: PLoS Genet. 2024 Mar 11;20(3):e1011196. doi: 10.1371/journal.pgen.1011196 (PMC10957079; doi:10.1371/journal.pgen.1011196)
Supplement: S2 Table — (DOCX) [file pgen.1011196.s005.docx]

**S2 Table. The putative AaERR binding sequence in the promoter region of the CM and LM key enzyme genes in *Ae. aegypti*.**

| **Gene name** | **AaERR binding sequence**  **(Red represent conserved sequence)** |
| --- | --- |
| GPI | AGACATAAGAAAAAGGTCAGTAGAAAAAAG |
| PYK | CTGACATACCCTAAGGTCCCAAAAAAACGA |
| PGM | GATCGAGTTCCAAAAGTCACGATTTTCACAA |
| PFK | TTGTTATACTCTTGACCTAGTCTTCAATTT |
| FAS | TGTAGTTTGCAAAGGTCATATCGTAACTAT |
| ACSL | GCACATCACCCTAAGGTCGAAAATATTATT |
| ACACA | GTAAACGTATACAAGGTCGATCTGCTTATT |
