# Supplementary material for: Ecdysone-controlled nuclear receptor ERR regulates metabolic homeostasis in the disease vector mosquito Aedes aegypti
Source: PLoS Genet. 2024 Mar 11;20(3):e1011196. doi: 10.1371/journal.pgen.1011196 (PMC10957079; doi:10.1371/journal.pgen.1011196)
Supplement: S3 Table — KD, knock down; FBs, fat bodies. (DOCX) [file pgen.1011196.s006.docx]

**S3 Table. Detailed presentation of two-tailed statistical test results and effect outputs.** KD, knock down; FBs, fat bodies.

| **Fig** | **Comparison** | **Statistical test** | **Effect Test Outputs** |
| --- | --- | --- | --- |
| **1A** | *AaERR* expression at each timepoint (FBs) | Mann-Whitney test;  Welch’s t test;  Unpaired t test;  Unpaired t test;  Unpaired t test;  Unpaired t test;  Unpaired t test;  Welch’s t test;  Welch’s t test | 72 h PE vs 6 h PE: p=0.0571  72 h PE vs 6 h PBM: p=0.1213  72 h PE vs 18 h PBM: p=0.0062  72 h PE vs 24 h PBM: p=0.0076  72 h PE vs 36 h PBM: p<0.0001  72 h PE vs 48 h PBM: p=0.0042  72 h PE vs 60 h PBM: p=0.0044  72 h PE vs 72 h PBM: p=0.0241  72 h PE vs 96 h PBM: p=0.0307 |
| **1B** | *AaERR* expression upon 20E treatment (FBs) | Welch’s t test | p=0.0018 |
| **1C** | *AaERR* expression after *EcR* KD (FBs) | Welch’s t test | p<0.0001 |
| **2A** | Fluc/Rluc activity after addition of EcR/USP or 20E-EcR/USP | Welch’s t test;  Unpaired t test | EcR/USP vs pAc5.1b: P=0.0176  20E-EcR/USP vs EcR/USP: p=0.0002 |
| **2C** | Enrichment of *AaERR* promoter fragments observed in ChIP assays involving EcR/USP proteins | Unpaired t test | *AaERR*: p<0.0001 |
| **2D** | Control samples in ChIP assays with EcR/USP proteins (*AaERR*) | Unpaired t test | *AaERR*: p<0.0001 |
| **3B** | Follicle size after *AaERR* KD | Mann-Whitney test | p<0.0001 |
| **3C** | Egg number after *AaERR* KD | Mann-Whitney test | p=0.0004 |
| **3D** | *Vg* expression after *AaERR* KD (FBs) | Welch’s t test | p=0.0015 |
| **4A** | Glucose, fructose and trehalose contents after *AaERR* KD (FBs) | Welch’s t test;  Unpaired t test;  Unpaired t test | Glucose: p=0.0005  Fructose: p=0.0054  Trehalose: p<0.0001 |
| **4C** | Glycogen levels after *AaERR* KD (FBs) | Welch’s t test | p=0.0008 |
| **4E** | Size of lipids after *AaERR* KD (FBs) | Unpaired t test | p<0.0001 |
| **4F** | TAG levels after *AaERR* KD (FBs) | Welch’s t test | p<0.0001 |
| **6A** | *GPI* and *PYK* expression after *AaERR* KD (FBs) | Unpaired t test;  Welch’s t test | *GPI*: p<0.0001  *PYK*: p=0.0063 |
| **6C** | Fluc/Rluc activity after addition of AaERR | Unpaired t test;  Unpaired t test | *GPI*: p<0.0001  *PYK*: p<0.0001 |
| **6E** | Enrichment of *GPI* and *PYK* promoter fragments observed in ChIP assays involving AaERR protein | Unpaired t test  Welch’s t test | *GPI*: p<0.0001  *PYK*: p=0.0192 |
| **6F** | Control samples in ChIP assays with AaERR protein (*GPI* and *PYK*) | Unpaired t test  Welch’s t test | *GPI*: p<0.0001  *PYK*: p=0.0058 |
| **7A** | *FAS* expression after *AaERR* KD (FBs) | Welch’s t test | p=0.0080 |
| **7B** | Fluc/Rluc activity after addition of AaERR | Welch’s t test | p=0.0021 |
| **7D** | Enrichment of *FAS* promoter fragments observed in ChIP assays involving AaERR protein | Welch’s t test | *FAS*: p=0.0065 |
| **7E** | Control samples in ChIP assays with AaERR protein (*FAS*) | Welch’s t test | *FAS*: p=0.0434 |
| **S1A** | *Met* expression after *Met* KD (FBs) | Unpaired t test | p<0.0001 |
| **S1B** | *AaERR* expression after *Met* KD (FBs) | Unpaired t test | p<0.0001 |
| **S1C** | *EcR* expression after *EcR* KD (FBs) | Unpaired t test | P=0.0005 |
| **S1D** | *AaERR* expression after *AaERR* KD (FBs) | Welch’s t test | p<0.0001 |
| **S3A** | *PFK* and *PGM* expression after *AaERR* KD (FBs) | Unpaired t test;  Unpaired t test | *PFK*: p<0.0001  *PGM*: p<0.0001 |
| **S3B** | *ACSL* and *ACACA* expression after *AaERR* KD (FBs) | Unpaired t test;  Mann-Whitney test | *ACSL*: p<0.0001  *ACACA*: p=0.0286 |
| **S3C** | Enrichment of promoter fragments from down-regulated CM genes in ChIP assays involving AaERR protein | Unpaired t test  Unpaired t test | *PFK*: p<0.0001  *PGM*: p<0.0001 |
| **S3D** | Enrichment of promoter fragments from down-regulated LM genes in ChIP assays involving AaERR protein | Unpaired t test  Unpaired t test | *ACSL*: p<0.0001  *ACACA*: p<0.0001 |
| **S3E** | Control samples in ChIP assays with AaERR protein (CM genes) | Welch’s t test  Unpaired t test | *PFK*: p=0.0075  *PGM*: p=0.0008 |
| **S3F** | Control samples in ChIP assays with AaERR protein (LM genes) | Welch’s t test  Unpaired t test | *ACSL*: p=0.001  *ACACA*: p=0.0162 |
