# Supplementary material for: Ecdysone-controlled nuclear receptor ERR regulates metabolic homeostasis in the disease vector mosquito Aedes aegypti
Source: PLoS Genet. 2024 Mar 11;20(3):e1011196. doi: 10.1371/journal.pgen.1011196 (PMC10957079; doi:10.1371/journal.pgen.1011196)
Supplement: S4 Table — ACLY, ATP citrate (pro-S)-lyase; MDH1, malate dehydrogenase; MDH2, malate dehydrogenase; TPI, triosephosphate isomerase; CHK, choline/ethanolamine kinase; IDH, isocitrate dehydrogenase; MINPP1, inositol-polyphosphate phosphatase; INO1, inositol-3-phosphate synthase. (DOCX) [file pgen.1011196.s007.docx]

**S4 Table. Transcriptomic changes of differentially expressed CM and LM genes in iAaERR mosquitoes.** ACLY, ATP citrate (pro-S)-lyase; MDH1, malate dehydrogenase; MDH2, malate dehydrogenase; TPI, triosephosphate isomerase; CHK, choline/ethanolamine kinase; IDH, isocitrate dehydrogenase; MINPP1, inositol-polyphosphate phosphatase; INO1, inositol-3-phosphate synthase.

| Metabolism | Pathway | Gene name | iAaERR VS iGFP | AaERR binding site |
| --- | --- | --- | --- | --- |
| Carbohydrate metabolism | Citrate cycle (TCA cycle) | ACLY | Down | No |
|  |  | DLAT | Down | No |
|  |  | MDH1 | Down | tcttctatcaatAAGGTCtaacatggttgt |
|  |  | MDH2 | Down | acgtcaacaacgAGGTCAtcgcactctatt |
|  | Glycolysis / Gluconeogenesis | GPI | Down | agacataagaaaAAGGTCAgtagaaaaaag |
|  |  | DLAT | Down | No |
|  |  | PFK | Down | aatatctttaagAAGGTCcatctgctcatt |
|  |  | ALDO | Down | No |
|  |  | TPI | Down | No |
|  |  | GAPDH | Down | ttccattcggaaTGACCTTtggaactttttt |
|  |  | PGK | Down | tatgcggttttgAAGGTCttggagtcaaac |
|  |  | ENO | Down | gcgtttcaataaGACCTAgatggttttgca |
|  |  | PYK | Down | ctgacataccctAAGGTCccaaaaaaacga |
|  |  | PGM | Down | acagatgcgagcGACCTTcacgcgacattc |
|  |  | LDH | Down | tgtgcagccgaaAAGGTCAccttcaacatct |
|  |  | ALDH1A | Down | aacatacgaaaaTGACCTgctaaagctgca |
|  |  | PGAM | Down | caatccttttgtTGACCTTgccactgtttaa |
|  | Pentose phosphate pathway | PGM | Down | acagatgcgagcGACCTTcacgcgacattc |
|  |  | TAL | Down | No |
|  |  | RPE | Down | ctgacggtcacgAAGGTCAtcagcgttttgc |
|  |  | TKT | Down | No |
|  |  | PFK | Down | aatatctttaagAAGGTCcatctgctcatt |
|  |  | GLD | Down | No |
|  |  | ALDO | Down | No |
|  |  | G6PD | Down | No |
|  |  | GPI | Down | agacataagaaaAAGGTCAgtagaaaaaag |
| Lipid metabolism | Glycerolipid metabolism | ALDH1A | Down | aacatacgaaaaTGACCTgctaaagctgca |
|  | Glycerophospholipid metabolism | CHK | Down | tataacggcagaTGACCTgtcttgaatgag |
|  | Inositol phosphate metabolism | TPI | Down | No |
|  |  | INO1 | Up | tattaaaaagtaAAGGTCtgttagtctcca |
|  |  | MINPP1 | Up | tcttaattgctcAGGTCAttgttggaatga |
|  | Peroxisome | ACSL | Down | actcgaatcgacAGGTCAaattaagccgtt |
|  |  | IDH | Up | agtaaccaccaaGACCTTtgtcgtgaattt |
|  | Fatty acid metabolism | ACADVL | Down | gttaagatcactAAGGTCgaacttttgccc |
|  |  | ACACA | Down | ccaaagtacctaAAGGTCAagaagtcaccga |
|  |  | FAS | Down | tgtagtttgcaTGACCTTtatcgtaactat |
|  |  | ACSL | Down | actcgaatcgacAGGTCAaattaagccgtt |
