# Supplementary material for: Ecdysone-controlled nuclear receptor ERR regulates metabolic homeostasis in the disease vector mosquito Aedes aegypti
Source: PLoS Genet. 2024 Mar 11;20(3):e1011196. doi: 10.1371/journal.pgen.1011196 (PMC10957079; doi:10.1371/journal.pgen.1011196)
Supplement: Attachment — Submitted filename: Response to Reviewers comments.docx [file pgen.1011196.s009.docx]

**Author's Responses to Reviewers**

Dear Editor,

We sincerely extend our thanks for you and reviewers insightful and constructive comments which would substantially improve our manuscript. We have carefully studied your comments and successfully completed all the major experiments requested by you. Our newly obtained data are presented in Figures 1, 2, 6, 7, S3 and Tables S1, S3, S4. We have also substantially revised our manuscript to fully address the questions.

We have responded to each comment point by provided the line numbers the place where the issue is addressed in the manuscript.

We hope that the correction will meet the standard for publication in PLOS Genetics and appreciate your kind consideration.

**Reviewer #1's Questions and Our Responses**

**Question 1:** In this study, Gen et al. delved into the functional implications of the estrogen-related receptor (ERR) in the regulation of metabolism in adult female mosquitoes following a blood meal. Their tissue culture and RNAi experiments demonstrated that the upregulation of ERR in blood-fed mosquitoes was triggered by the elevation of 20E after blood feeding. Notably, the knockdown of ERR via RNAi in female mosquitoes elicited significant shifts in various carbohydrate and lipid metabolites, accompanied by significant changes in the expression of key genes involved in these metabolic pathways, resulting in a decrease in mosquito fecundity. This study established ERR as a key player of the 20E regulatory cascade, directly modulating metabolic flux during mosquito reproduction. While the regulatory function of ERR in metabolic homeostasis has been documented in other insect species, this study is particularly significant due to its focus on the metabolic role of ERR in the context of 20E signaling in a vital disease vector. The findings of this study provide significant advancement in the understanding of how metabolism is mobilized to meet the energy demands for mosquito adult reproduction. The conducted experiments were meticulously designed, incorporating appropriate controls and rigorous statistical analyses.

However, an important concern arises regarding the study's emphasis on genes positively regulated by ERR, potentially neglecting those that may be negatively regulated. To provide a more comprehensive understanding, it is imperative to investigate whether any of the 118 upregulated genes following ERR knockdown are associated with metabolism. Additionally, exploring potential ERR binding sites within these upregulated genes would significantly contribute to a more balanced interpretation of the underlying molecular mechanisms.

**Response:** Among the 118 upregulated genes following *AaERR* knockdown, we found that three genes were involved in carbohydrate metabolism or lipid metabolism, including the genes encoding isocitrate dehydrogenase (IDH), inositol-polyphosphate phosphatase (MINPP1) and inositol-3-phosphate synthase (INO1). Then, we predicted the potential binding motifs of AaERR in the promoter regions of these three genes. As shown in **S4 Table**, the promoters of all these three genes have AaERR binding sites, however, the subsequent ChIP-qPCR experiments verified that only the promoter region of *IDH* could be enriched by AaERR. The results are presented as follows. Therefore, although three upregulated genes are associated with metabolism, only *IDH* is regulated by AaERR. Furthermore, as shown in S4 Table, Fig 2C-2D, Fig 6E-6F, Fig 7D-7E and S3C-F Fig, most metabolism-related genes are downregulated by AaERR.

**Question 2:** Line 85. Rephrase this sentence to enhance clarity.

Line 85. “This indicates that mosquitoes meet energy requirements for blood feeding and oocyte maturation.”

**Response:** We have made the following corrections to the sentence to make it clearer.

**Line 83.** “Thus, a significant accumulation of metabolites during the JH-regulated PE phase was utilized later for vitellogenesis.”

**Question 3:** Fig. 1D. Specify the time point for the measurement of the ERR transcript after the knockdown of EcR.

**Response:** To provide clarity, we have detailed the specific time point for measuring the *AaERR* transcript levels following *EcR* knockdown in the revised legend of Figure 1C (Former Fig. 1D).

**Line 162.** “Mosquitoes were given a blood meal three days after *EcR* knockdown, and FBs were collected to measure the *AaERR* transcript at 36 h PBM.”

**Question 4:** Fig. 2. Rearrange the legend to place the description of error bars after the statistical data for improved comprehension.

**Response:** We have placed the description of the error bars immediately follows the presentation of the statistical data.

**Question 5:** EMSA results (Figs. 2, 6, and 7). Rectify the description of V5 antibody and the labeled AaERR (cold) probe as negative controls while the mutant probe and IgG as positive controls. They were introduced to validate the specific binding of AaERR to its binding site, not as experimental controls.

**Response:** We have made the necessary corrections in our manuscript as per your suggestions. The revised sentences are as follows:

**Fig 2. Results** **Section.**

**Line 234.** Revised Text:

“The shift band was evident after the biotin-labeled *AaERR* probe was combined with the nuclear extract. This band was not visible after the addition of 50 × molar excess of the unlabeled specific *AaERR* probe to the binding reaction, whereas the addition of 50 × molar excess of the unlabeled mutant probe did not have this effect. Moreover, the band shift disappeared from a gel when the reaction mixture was pre-incubated with the anti-V5 antibody for 1-2 h at 4°C. In contrast, when the anti-IgG antibody was added to the mixture instead of the anti-V5 antibody, the gel band remained indicating that this antibody did not disrupt the specific probe-protein interaction (Fig 2B).”

**Fig 2. Figure legend.**

**Line 214.** Revised Text:

“The unlabeled *AaERR* probe and its corresponding mutant probe, both in 50 × molar excess, were employed to verify the binding site’s specificity. The anti-V5 antibody was utilized to confirm the binding specificity of antibody with the EcR/USP-V5 complex in protein-probe binding mixtures, with the binding efficacy demonstrated using the anti-IgG antibody.”

**Fig 6. Results** **Section.**

**Line 432.** Revised Text:

“The band was apparent after the biotin-labeled *GPI* or *PYK* probes was combined with the nuclear extract containing plasmid. It disappeared after adding 50 × molar excess of unlabeled specific probes. However, the band remained after the 50 × molar excess unlabeled mutant probe was added to the binding reaction. To further confirm the presence of AaERR-V5 in the nucleoprotein-probe complexes, the anti-V5 antibody and nuclear extract were incubated together on ice for 1-2 h before the binding experiment, then the band shift disappeared. However, the addition of anti-IgG antibody did not affect the shift band indicating that this antibody did not disrupt the specific probe-protein interaction (Fig 6D).”

**Fig 6. Figure legend.**

**Line 398.** Revised Text:

“The anti-V5 antibody was employed to confirm the binding of AaERR-V5 protein to the biotin-labeled probe, while the anti-IgG antibody served to demonstrate the specific interaction between the anti-V5 antibody and the AaERR-V5 protein. To ascertain the binding motifs’ specificity, we used the unlabeled *GPI* and *PYK* probes, and its mutant form, each in 50 × molar excess.”

**Fig 7. Results Section.**

**Line 502.** Revised Text:

“When the biotin-labeled *FAS* probe combined with nucleoprotein overexpressed AaERR-V5 protein, the specific complex band appeared in the gel lane. And this band disappeared after adding the 50 × molar excess competitive unlabeled probe to the labeled probe and nucleoprotein mixture. The AaERR-V5 nucleoprotein was incubated with the anti-V5 antibody on ice for 1-2 h before being mixed with the labeled probe, which resulted in the disappearance of the band shift. Contrary to the addition of the unlabeled probe, the band was notably observed again following the addition of the unlabeled mutated probe. A similar band shift was observed when the anti-IgG antibody was incubated with the nucleoprotein sample, confirming the specificity binding of AaERR-V5/probe and anti-V5 antibody (Fig 7C).”

**Fig 7. Figure legend.**

**Line 486.** Revised Text:

“The anti-V5 antibody was used to verify the binding of AaERR-V5 to the biotin-labeled probe, with the anti-IgG antibody further confirming the specific binding between the anti-V5 antibody and the protein. The specificity of the binding site was demonstrated using the unlabeled *FAS* probe and its mutant form, both in 50 × molar excess.”

**Question 6:** Line 180. Correct the terminology to reflect that the reporter assay measured luminescence, not fluorescence.

**Response:** According to your suggestion, we revised the manuscript to correctly describe the reporter assay measurements as luminescence.

**Question 7:** Line 251. Clarify that the qPCR measured only 4 genes, not all genes.

**Response:** We have amended the manuscript to accurately reflect that our qPCR analysis was conducted on four specific genes, not all genes.

The revised text in **Line 372** now: “Consistent with the metabolic phenotype observed above, the mRNA levels of four representative genes, including *glucose-6-phosphate isomerase* (*GPI*), *pyruvate kinase* (*PYK*), *phosphofructokinase* (*PFK*), and *phosphoglucomutase* (*PGM*), were significantly lower in iAaERR mosquitoes (Figs 6A and S3A).”

**Question 8:** Lines 279-280. Provide a precise description that the addition of anti-IgG did not affect the probe-protein interaction or the appearance of the shift band.

**Response:** Please see the answer to question 5 (Line 438).

**Question 9:** Line 300. Specify that nuclear proteins were preincubated with the anti-V5 antibody for clarity.

**Response:** Please see the answer to question 5 (Line 506).

**Question 10:** Lines 301-304. Elaborate on the opposite effect observed, providing explicit details to avoid ambiguity.

**Response:** Please see the answer to question 5 (Line 508).

**Question 11:** Line 309. The statement is inaccurate. Not all members of the 20E regulatory cascades are nuclear receptors.

**Response:** Thank you for highlighting the need for accuracy in our description on Line 309. We have revised this section of the manuscript to address the inaccuracy pointed out.

**Line 523.** The updated sentence is: “In mosquitoes, 20E plays a major role in regulating PBM reproductive events, and its action is mediated by the cascade of transcription factors, many of which belong to the nuclear receptor family.”

**Question 12:** Fig 5. Explain the concept of Gene Ratio to facilitate better comprehension.

**Response:**

**Line 356.** We have revised the legend: “Gene ratio is defined as the ratio of the number of genes enriched from each category to the total number of genes analyzed in the KEGG pathway enrichment analysis.”

**Question 13:** Consider adding a dataset to report the transcriptomic changes of annotated CM and LM genes in iAaERR mosquitoes to provide additional context and support.

**Response:** We have included a new dataset in **S4 Table** of our manuscript, which details the transcriptomic changes in annotated CM and LM genes in iAaERR mosquitoes. Additionally, we also incorporated information about the AaERR binding motifs predicted in our study in S4 Table.

**Question 14:** Provide an explanation of how the downregulation of very long-chain acyl-CoA dehydrogenase (ACADVL), an enzyme required for fatty acid oxidation, fits into the observed decrease in the levels of TAG in iAaERR mosquitoes.

**Response:** We have included a detailed explanation in the manuscript regarding the interplay between *ACADVL* downregulation and the observed decrease in TAG levels in iAaERR mosquitoes.

**Line 452.** The revised section: “Our transcriptome analysis revealed four DEGs related to fatty acid metabolism in *AaERR* RNAi knockdown mosquitoes (Fig 5C). Despite the downregulation of all four genes in these mosquitoes, three are involved in fatty acid synthesis, and one, *very long-chain acyl-CoA dehydrogenase* (*ACADVL*), is associated with fatty acid degradation. This pattern indicates a possible pronounced inhibition of the fatty acid synthesis pathway compared to the degradation pathway in iAaERR mosquitoes, leading to an overall reduction in lipid levels. Therefore, the three enzymes associated with fatty acid synthesis attracted a particular interest in our study.”

**Reviewer #2's Questions and Our Responses**

Working on the mosquito Aedes aegypti, Geng and colleagues found that ERR knockdown resulted in deficient phenotypes of ovary as well as the metabolism of carbohydrates and lipids during the gonadotropic cycle. ERR transcription was directly activated by 20E via EcR binding to the EcRE in ERR promoter. They also found that ERR directly activated the expression of metabolic genes GPI, PYK and FAS by binding to the corresponding ERR-responsive motif in the promoters of these genes. Overall, the study represents a new and interesting finding in hormonal regulation of female reproduction. The data are comprehensive and presented in a logical sequence. The manuscript is well written. However, there are some concerns that need to be addressed.

**Response:** We would like to thank you for the valuable comments and suggestions, and considering our results interesting and logical.

**Question 1:** The developmental profile of **20E titer** could be added in Fig 1A.

**Response:** We have incorporated the developmental profile of 20-hydroxyecdysone (20E) titer in Figure 1A and provided an accompanying description in the manuscript. The schematic representing the fluctuation of the titers of 20E during the mosquito reproduction was shown in Fig. 1A was modified from the paper (PMID: 1059110).

**Figure 1A.**

**Question 2:** ERR distributes in both cytoplasm and nuclei from PBM 24h to 48h, but mainly in nuclei at 72h (Fig 1B). Authors should discuss the dynamic of subcellular localization of ERR, as well as its relation with ERR regulation of carbohydrate and lipid metabolism during the mosquito reproduction.

**Response:** Upon serious consideration, we decided to exclude Figure 1B from our manuscript. We recognized a disconnect between the mRNA expression profiles and the corresponding protein distribution dynamics. We hypothesized that the dynamic distribution of AaERR is associated with the regulation of its target genes involved in carbohydrate and lipid metabolism during mosquito reproduction. However, upon reflection, we consider that the data presented in Fig 1B are insufficient to conclusively support this hypothesis. To prevent potential confusion and ensure the clarity of our findings, we have decided to omit this figure from our revised manuscript.

**Question 3:** Please indicate pAc5.1b-USP-V5 in Fig 2 and its legend.

**Response:** We have now clearly indicated pAc5.1b-USP-V5 in both the figure and its legend.

**Question 4:** The tissues (fat body or hemolymph) should be addressed for glucose, fructose, and trehalose measurement in Fig 4A and4 C as well as the Results and figure legends.

**Response:** We have clarified in both the results section and the legends of Figures 4A and 4C that the glucose, fructose, trehalose, and glycogen measurements were specifically conducted in the fat body tissues.

**Question 5:** Among ME genes, ERR directly bound to the promoters of GPI and PYK and activated their expression. How about other CM pathway genes like PFK and PGM? Authors should discuss the potential regulation of GPI and PYK by ERR.

**Response:** We prepared a Table to show that most ME genes have the AaERR binding motif in their promoter regions (S4 Table). And in this work, we pick *GPI* and *PYK* as the representative genes to explore the direct binding between AaERR and the promoters experimentally. In fact, other ME genes like *PFK* and *PGM* have the potential binding. To prove this, we conducted ChIP-qPCR experiments to examine the interactions of AaERR with the promoters of *PFK* and *PGM*. Our results confirmed AaERR binding to these regions, as presented in **S3C Fig**.

**S3C Fig**

**Question 6:** Similarly, ERR directly bound to the promoter of FAS and activated its expression, whereas ACSL and ACACA were not directly regulated by ERR. This should be discussed too.

**Response:** We performed ChIP-qPCR experiments to investigate the potential interactions between AaERR and the promoters of *ACSL* and *ACACA*. Our findings, displayed in **S3D Fig**, indicate that AaERR indeed binds to the promoter regions of both *ACSL* and *ACACA*.

**S3D Fig**

**Question 7:** Additional bands were seen when IgG was present in EMSA of Fig 6D but not Fig 7C. In addition to luciferases assay and EMSA, ChIP could be a good plus to investigate the binding of EcR/USP and ERR to the promoters.

**Response:**

1. Regarding the EMSA in Figure 6D, the additional bands observed when anti-IgG antibody is present might be attributed to differences in probe interactions with nucleoproteins. We hypothesize that the anti-IgG antibody may slightly affect the binding of some probe to proteins, leading to extra bands beneath the band shift. However, the position of the primary band shift remains identifiable based on other lanes’ results.
2. Following your recommendation, we performed ChIP-qPCR experiments to confirm the binding of EcR/USP and AaERR to the promoters of their downstream genes. These results are illustrated in **Figures 2C**, **2D**, **6E**, **6F**, **7D, 7E**, and **S3C, S3D, S3E, S3F Fig**.

**Fig 2C, Fig 2D.**

**Fig 6E, Fig 6F.**

**Fig 7D, Fig 7E.**

**S3C Fig, S3D Fig, S3E Fig and S3F Fig.**

**The source data for these findings are provided in attachment accompanying the manuscript.**
